# Nasal colonization with methicillin-resistant *Staphylococcus aureus* among elderly living in nursing homes in Brazil: risk factors and molecular epidemiology

**DOI:** 10.1186/s12941-018-0271-z

**Published:** 2018-05-04

**Authors:** Monica da Silveira, Maria de Lourdes Ribeiro de Souza da Cunha, Camila Sena Martins de Souza, Adriana Aparecida Feltrin Correa, Carlos Magno Castelo Branco Fortaleza

**Affiliations:** 1 Department of Tropical Diseases, Botucatu School of Medicine, University Hospital, UNESP-Univ Estadual Paulista, Botucatu, SP Brazil; 2 Department of Microbiology and Immunology, Botucatu Biosciences Institute, UNESP-Univ Estadual Paulista, Botucatu, SP Brazil

**Keywords:** *Staphylococcus aureus*, MRSA, Nursing homes

## Abstract

**Background:**

Methicillin-resistant *Staphylococcus aureus* poses a threat to elderly living in nursing homes. Studies focusing on the epidemiology of colonization may help in the design of infection control strategies.

**Objective:**

To identify factors associated with MRSA colonization and the dissemination of clones among nursing home residents.

**Methods:**

Nasal swabs were collected from 300 persons from nine nursing homes in the city of Bauru, Brazil. Resistance to methicillin was identified through amplification of the *mec*A gene. Strain typing (Pulsed-Field Gel Electrophoresis) and characterization of the *Staphylococcal Chromosome Cassette* (SCC) *mec* was performed. Univariate and multivariable models were used to identify predictors of overall *S. aureus* and MRSA carriage.

**Results:**

Rates of *S. aureus* and MRSA colonization were 17.7 and 3.7%, respectively. Age and recent admission to a hospital were independently associated with colonization with *S. aureus*. MRSA colonization was associated with living in small (< 15 residents) and medium-sized (15–49 residents) facilities, as well as with recent hospitalization. Most MRSA strains carried SCC*mec* types II or IV, and there was evidence of clonal spread within and among different facilities.

**Conclusions:**

MRSA may be introduced in nursing homes form hospitals or arise from the community setting. Screening for asymptomatic colonization may identify persons with greater risk for infection, and is advised for residents discharged from acute care hospitals.

**Electronic supplementary material:**

The online version of this article (10.1186/s12941-018-0271-z) contains supplementary material, which is available to authorized users.

## Introduction

Recent studies documented high prevalence and dissemination of colonization with methicillin-resistant *Staphylococcus aureus* (MRSA) within long-term care facilities—especially those caring for elderly persons [1, 2]. This phenomenon is a challenge for infection control [3, 4], and poses a special threat for the residents, for several reasons. First, current evidence suggests that person MRSA-colonized persons are more likely to develop invasive infections than those not colonized or those carrying methicillin-susceptible *S. aureus* [5]. The elderly are a group not only more susceptible to infection [6], but also at higher risk of adverse events related to antimicrobial therapy (e.g., vancomycin-related nephropathy) [7]. Colonized persons contribute to the dissemination of MRSA strains within nursing homes [4]. Finally, the emergence of Community-Associated MRSA (CA-MRSA) provides new routes for introduction of virulent strains into those facilities [8].

While MRSA has been extensively studied in the United States and Europe, data from developing countries are scarce [9]. This gap is especially worrisome in regard to institutionalized elderly people. In this context, several factors, both cultural (affecting the magnitude and reasons for admittance to nursing homes) and organizational (including physical structure, staffing and care processes in the facilities) may interfere on MRSA epidemiology.

In Brazil, the population aged 65 or greater is continuously increasing, reaching 14 million in year 2010 (8.6% of total population) [10]. Institutionalization is still uncommon in that country, and estimates point out that only 0.8% of the elderly live in nursing homes [11]. A recent study identified female gender, advanced age (above 70 or 80), widowhood and lower schooling among predictors for institutionalization in a city in Southern Brazil [12]. The same study reported significantly less physical activity among that population. With regard to nursing homes in Brazil, they are either public, private or charitable, and the quality of care varies widely [13]. The peculiar characteristics of those nursing homes and their residents may contribute to the acquisition of MRSA colonization and/or infection. Our study aimed at quantifying the asymptomatic carriage of overall *S. aureus* and MRSA and at identifying factors related to this phenomenon. We also aimed at the identification of routes of introduction and dissemination of MRSA strains within facilities.

## Methods

### Study setting

The study was conducted in the city of Bauru, São Paulo State, Brazil (22**°**18′ 53″S, 49**°**03′ 38″W). The city has approximately 370,000 inhabitants in year 2017, 9.1% of whom are aged 65 or greater. The city health department estimates that approximately 700 elderly persons live in 16 nursing homes.

### Study design and subjects

The study had a cross-sectional design. We included residents from nine nursing homes. The inclusion criteria were length-of-stay in the nursing home of more than 30 days and agreement to participate in the study. There were no specific exclusion criteria.

### Data collection

Data were collected on interviews with the elders and their caregivers, and complemented with chart reviews. We recorded data on demographics, comorbidities (including the Charlson comorbidity index) [14] and functional impairment (measured by the Karnofsky scale) [15]. Other collected data included length-of-stay in the facility, hospital admission in the past 6 months, invasive procedures (surgeries and others), recent infections and use of antimicrobials.

### Microbiology and molecular methods

Nasal swabs were obtained from study subjects. Specimens were transported in Stuart medium and cultured in Baird Parker Medium. Species identification and disk diffusion tests (for Oxacillin and Cefoxitin) followed standard recommendations [16]. For the purposes of our study, *S. aureus* isolates were classified as MRSA when they harbored the *mecA* gene, which is responsible for methicillin-resistance. Polymerase chain reaction (PCR) for detection of *mecA* was performed as described by Murakami et al. [17]. The multiplex-PCR protocol described by Milheiriço et al. [18] was used for the characterization of the Staphylococcal Cassette Chromosome *mec* (SCC*mec*). Strain typing was performed using a protocol modified from McDoughal et al. [19]. Cluster analysis was performed on the basis of the Dice similarity coefficient, using BioNumerics 6.1 software (Applied Maths, Belgium).

### Statistical analysis of epidemiologic data

Data were stored in EPI INFO 7 (Centers for Diseases Control and Prevention, Atlanta, GA) and analyzed in SPSS 19.0 (IBM, Armonk, NY). In a first step, we arbitrarily classified facilities as small-sized (< 15 residents), medium-sized (15–49 residents) or large (50 or more residents). Those groups were compared in regard to residents’ morbidities and prevalence of the pathogens of interest. The second step involved univariate analysis of individual factors associated with *S. aureus* or MRSA colonization. Categorical data were analyzed using the Chi square or Fisher exact test, while continuous data were compared using the Mann–Whitney U test. In the third and final step, we applied multivariable models of logistic regression in order to identify independent predictors for both outcomes. A backward process was applied for selection of variables. The criteria for inclusion and exclusion of variables in the models were P values of 0.05 and 0.1, respectively [20].

## Results

We included 300 subjects in the study. The distribution of subjects according to the size of facilities was: large (A and F), 169; medium-sized (E and I), 68; small-sized (B, C, D, G, H), 63. The prevalence of colonization with *S. aureus* and MRSA were 17.7 and 3.7%, respectively.

When compared to residents from large nursing homes, those in small-sized facilities had higher scores in the Charlson index (median 2 [Interquartile range (IQR), 2–3] versus 1 [IQR 0–2], *P *< 0.001), while those in medium-sized institutions presented lower values in the Karnofsky scale [median 55 (IQR, 40–70] versus 60 [IQR, 50–80], *P *= 0.041). The prevalence of *S. aureus* carriage was higher in small-sized facilities (25.4% versus 13.0%, *P *= 0.039). On the other hand, both medium (7.4%) and small (7.9%) institutions had significantly higher rates of MRSA colonization, when compared to large ones (0.6%).

The univariate analysis identified similar predictors for overall *S. aureus* and MRSA colonization: age, small-sized facilities and recent admission to acute care hospitals. In the multivariable models, age and recent hospitalization were associated with *S. aureus* carriage, while living in small-sized or medium-sized institutions and recent admittance to a hospital predicted MRSA colonization. Table 1 presents results of the final multivariable model, while detailed univariate analysis is available in Additional files 1, 2.

**Table 1:** Multivariable analysis of factors associated with overall *Staphylococcus aureus* and MRSA colonization among elderly persons living in nursing homes in Bauru, Brazil

Risk factors	Coefficient (beta)	Standard error	OR (95% CI)	*P*
*Staphylococcus aureus*				
Age	0.031	0.014	1.03 (1.00–1.06)	0.029
Recent hospital admission	1.388	0.430	4.01 (1.73–9.31)	0.001
MRSA				
Medium-sized nursing home^a^	2.404	1.131	11.07 (1.21–101.69)	0.034
Small-sized nursing home^a^	2.420	1.132	11.25 (1.22–103.46)	0.033
Recent hospital admission	2.284	0.706	9.82 (2.46–39.18)	0.001

300 subjects were included. Overall *Staphylococcus aureus* colonization was found in 53 subjects, while MRSA colonization was found in 11 subjects

*MRSA* Methicillin-resistant *Staphylococcus aureus*, *OR* odds ratio, *CI* confidence interval

^a^ Versus large nursing homes

**Fig. 1:**
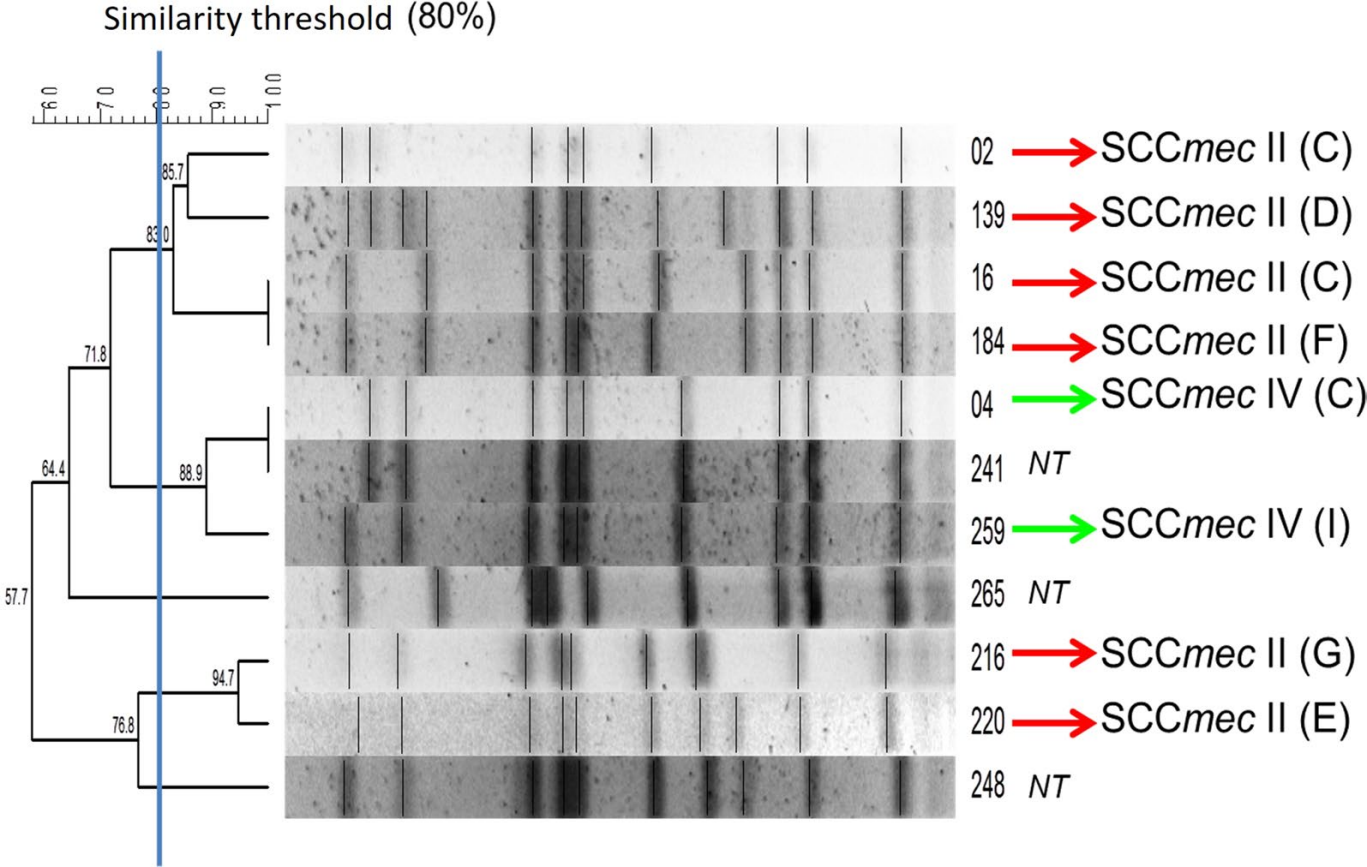
Dendrogram presenting results from strain typing analysis (Pulsed-Field Gel Electrophoresis), alongside with the characterization of the Staphylococcal Chromosome Cassette *mec*. Letters in parenthesis indicate the nursing homes where MRSA carriers lived. A similarity cutoff of 80% in Dice Similarity Index was applied to define clones

In the molecular analysis (Fig. 1), six out of 11 MRSA isolates harbored the SCC*mec* type II, while two isolates harbored SCC*mec* type IV. The SCC*mec* from remaining strains was not typeable with the Milheiriço protocol. Strain typing with PFGE characterized a major clone comprising four isolates from facilities C (2), D (1) and F (1). Two other clones were identified, comprising three (from C, E and I) and two (from G and E) isolates.

## Discussion

Nursing homes have been regarded as reservoirs of MRSA and sources of dissemination of strains for acute care hospitals [1]. Prevalence rates reported in previous studies vary from zero to more than 20% [2]. In this context, our pooled prevalence rate (nearly 4%) characterizes a picture of moderate danger. However, some findings deserve attention.

Both the prevalence of overall *S. aureus* and MRSA were higher in small-sized institution. The most striking contrast was found between facility A (155 residents, no MRSA) and C (three out of 16 residents carrying MRSA). In fact, this latter institution presented several deficiencies in staffing and structure, and was closed by sanitary authorities weeks after our visit. However, other small-and medium-sized nursing homes presented similar deficiencies in structure and healthcare assistance. Briefly, those facilities had less visits from medical doctors (usually every 2 weeks, while in large nursing homes doctors visited residents weekly or even more frequently). Also, physical activities were performed more often in large and medium-sized nursing homes than in small ones. Finally, residents in small-sized facilities were more frequently exposed to the same caregivers than those from large nursing homes (due to smaller staffing). Those aspects, together with greater severity and functional impairment among residents of small institutions, could contribute to the increased circulation of MRSA. Further investigation is needed to analyze the association between the sizes of facilities and other critical outcomes, such as invasive infection and death. Also, futures studies can perform a more detailed analysis of infection control measures in each facility. It is worth noting, however, that none of the nursing homes included in this study had formal infection control programs.

Other interesting data are provided in the joint analysis of epidemiological and molecular methods. Institutionalized elderly are often admitted to acute care hospitals, and this provides a two-way route for *S. aureus* and MRSA dissemination. In our study, 4 out of 6 MRSA isolates harboring SCC*mec* II were recovered from residents with a history of hospital admission in the past year. A previous report from a reference hospital in the same city where this study was conducted found extensive predominance of a SCC*mec* III clone among MRSA nosocomial isolates [21]. However, that clone—often termed the Brazilian epidemic clone [22]—is presently being replaced in Brazilian hospitals by SCC*mec* II-harboring MRSA [23].

On the other hand, two subjects were colonized by a single clone of SCC*mec* IV-harboring MRSA (one of whom reported recent hospital admission). Since that cassette is found in CA-MRSA, we cannot rule out the introduction of MRSA into nursing homes from the community. In fact, those long-term care facilities seem to be intermediary spaces—halfway between the community and hospitals.

Our study has some limitations, including the relatively small number of subjects and outcomes (especially MRSA colonization). Also, there are limitations inherent to the logistic regression analysis, which might overestimate associations. However, it also has strengths, including the number and representativeness of the nursing homes included in the study city, the extensive collection of individual-based data and performance of molecular typing.

The control of MRSA spread within nursing homes is challenging [3]. A systematic review focusing on that theme failed to identify effective measures, and concluded that this issue requires further research [24]. Even though those findings were disappointing, we believe that studies on the epidemiology of MRSA in different context can contribute to finding the best way for infection control. In this sense, our study points out to important items. Sanitary audits in small and medium-sized facilities, together with screening of residents returning after hospitalizations, implementation of barrier methods (such as cohorting of colonized persons) and overall strengthening of infection control measures may be key-points for MRSA control in Brazilian nursing homes.

## Additional files

**Additional file 1.** Flow-chart of inclusion of subjects in the study.

**Additional file 2: Table S1.** Factors associated to nasal carriage of *Staphylococcus aureus* among residents in nursing homes in Bauru, Brazil. **Table S2.** Factors associated to nasal carriage of MRSA among residents in nursing homes in Bauru, Brazil.
