# Supplementary figures and images for: Nasal colonization with methicillin-resistant Staphylococcus aureus among elderly living in nursing homes in Brazil: risk factors and molecular epidemiology
Source: Ann Clin Microbiol Antimicrob. 2018 May 4;17:18. doi: 10.1186/s12941-018-0271-z (PMC5934845; doi:10.1186/s12941-018-0271-z)

**SUPPLEMENTATY FILE (Flow-Chart of Inclusion of Subjects in the study)**


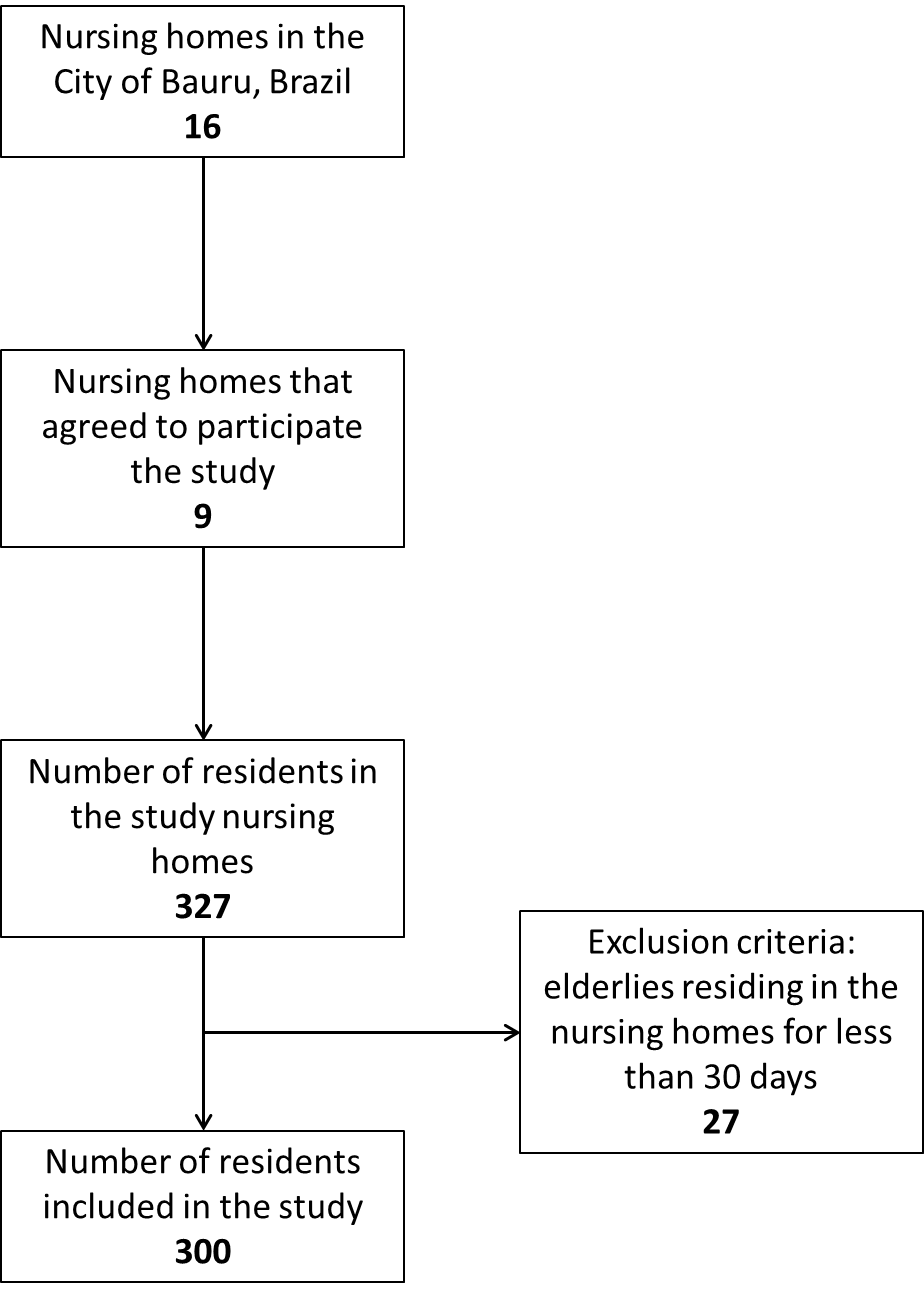

Supplement: Supplementary file 1 — Additional file 1. Flow-chart of inclusion of subjects in the study. [file 12941_2018_271_MOESM1_ESM.docx]
