# Supplementary material for: Nasal colonization with methicillin-resistant Staphylococcus aureus among elderly living in nursing homes in Brazil: risk factors and molecular epidemiology
Source: Ann Clin Microbiol Antimicrob. 2018 May 4;17:18. doi: 10.1186/s12941-018-0271-z (PMC5934845; doi:10.1186/s12941-018-0271-z)
Supplement: Supplementary file 2 — Additional file 2: Table S1. Factors associated to nasal carriage of Staphylococcus aureus among residents in nursing homes in Bauru, Brazil. Table S2. Factors associated to nasal carriage of MRSA among residents in nursing homes in Bauru, Brazil. [file 12941_2018_271_MOESM2_ESM.docx]

Table S1. Factors associated to nasal carriage of *Staphylococcus aureus* among residents in nursing homes in Bauru, Brazil.

|  | | **Univariate analysis** | | | | | | | **Multivariable analysis** | | | |  |
| --- | --- | --- | --- | --- | --- | --- | --- | --- | --- | --- | --- | --- | --- |
| **Risk Factors** | | ***S. aureus* (52)** | **Negative (n=248)** | | **OR (95%CI)** | | | ***P*** | **OR (95%CI)** | | ***P*** | |  |
| ***Size category*** | |  |  | |  | | |  |  | |  | |  |
| Large (reference) | | 23 (42.6) | 146 (59.3) | | 1.0 | | | … |  | |  | |  |
| Medium-sized | | 15 (27.8) | 53 (21.7) | | 1.76 (0.87-3.70) | | | 0.12 |  | |  | |  |
| Small-sized | | **16 (29.6)** | **47 (19.1)** | | **2.16 (1.05-4.43)** | | | **0.03** |  | |  | |  |
| ***Demographic data*** | |  |  | |  | | |  |  | |  | |  |
| Female gender | | 37 (68.5) | 145 (58.9) | | 1.52 (0.81-2.84) | | | 0.19 |  | |  | |  |
| Age, median (quartiles) | | **79 (70-87)** | **75 (66-82)** | | **…** | | | **0.01** | **1.03 (1.01-1.06)** | | **0.02** | |  |
| Months since admission, median (quartiles) | | 34.5 (12-85) | 48 (12-182) | | … | | | 0.13 |  | |  | |  |
| ***Medical data / comorbidities*** | |  |  | |  | | |  |  | |  | |  |
| Karnofsky index, median (quartiles) | | 50 (40-70) | 60 (50-80) | | … | | | 0.07 |  | |  | |  |
| Chalrson index, median (quartiles) | | 2 (1-3) | 2 (0-2) | | … | | | 0.82 |  | |  | |  |
| Heart disease | | 5 (9.3) | 22 (8.9) | | 1.04 (0.38-2.88) | | | 0.94 |  | |  | |  |
| Pulmonary disease | | 2 (3.7) | 10 (4.1) | | 0.91 (0.19-4.26) | | | 0.90 |  | |  | |  |
| Renal disease | | 1 (1.9) | 2 (0.8) | | 2.30 (0.20-25.86) | | | 0.48 |  | |  | |  |
| Diabetes mellitus | | 9 (16.7) | 44 (17.9) | | 0.91 (0.41-2.02) | | | 0.83 |  | |  | |  |
| CNS disease (other than dementia) | | 25 (46.3) | 95 (38.6) | | 1.37 (0.75-2.48) | | | 0.29 |  | |  | |  |
| Dementia | | 29 (53.7) | 156 (63.4) | | 0.67 (0.37-1.21) | | | 0.18 |  | |  | |  |
| Solid malignancy | | 3 (5.6) | 15 (6.1) | | 0.91 (0.25-3.25) | | | 0.87 |  | |  | |  |
| ***Exposure to healthcare**** | |  |  | |  | | |  |  | |  | |  |
| Admission to acute care hospitals | | **12 (22.2)** | **16 (6.5)** | | **4.10 (1.81-9.30)** | | | **<0.001** | **3.75 (1.63-8.61)** | | **0.002** | |  |
| Surgery | | 2 (3.7) | 13 (5.3) | | 0.68 (0.15-3.14) | | | 0.47 |  | |  | |  |
| Use of any antimicrobial | | 12 (22.2) | 55 (22.4) | | 0.99 (0.49-2.14) | | | 0.98 |  | |  | |  |
| Use of β-lactams | | 9 (16.7) | 24 (9.8) | | 1.85 (0.81-4.24) | | | 0.16 |  | |  | |  |
| Use of Quinolones | | 4 (7.4) | 24 (9.8) | | 0.74 (0.25-2.28) | | | 0.59 |  | |  | |  |
| Use of Macrolides | | 1 (1.9) | 10 (4.1) | | 0.44 (0.06-3.55) | | | 0.43 |  | |  | |  |
| Use of other antimicrobials | | 4 (7.4) | 9 (3.7) | | 2.11 (0.62-7.11) | | | 0.19 |  | |  | |  |
| Naso-enteral tube feeding | | 2 (3.9) | 6 (2.4) | | 1.54 (0.60-7.84) | | | 0.64 |  | |  | |  |
| Urinary catheter | | 3 (5.7) | 11 (4.5) | | 1.27 (0.34-0.70) | | | 0.72 |  | |  | |  |
| ***Recent infections**** | |  |  | |  | | |  |  | |  | |  |
| Skin/Soft tissue | | 8 (21.1) | 46 (17.6) | | 1.25 (0.5402.91) | | | 0.60 |  | |  | |  |
| Pneumonia | | 3 (5.7) | 51 (6.5) | | 0.85 (0.24-3.01) | | | 1.00 |  | |  | |  |
|  | | |  | |  |  | | |  | |  | |  |

**Note.** Data in number(%), except when specified. Significant results are presented in boldface. OR, Odds Ratio. CI, Confidence Interval, CNS, Central Nervous System.

*In the past year.

Table S2. Factors associated to nasal carriage of MRSA among residents in nursing homes in Bauru, Brazil.

|  | **Univariate analysis** | | | | | **Multivariable analysis** | | |
| --- | --- | --- | --- | --- | --- | --- | --- | --- |
| **Risk Factors** | **MRSA (11)** | **Negative (n=289)** | **OR (95%CI)** | ***P*** | **OR (95%CI)** | | ***P*** |  |
| ***Size category*** |  |  |  |  |  | |  |  |
| Large (reference) | 1 (9.1) | 168 (58.13) | 1.0 | … | 1.0 | |  |  |
| Medium-sized | **5 (45.5)** | **63 (21.8)** | **13.33 (1.53-116.22)** | **0.12** | **11.11 (1.21-102.17)** | | **0.03** |  |
| Small-sized | **5 (45.5)** | **58 (20.07)** | **14.48 (1.66-126.39)** | **0.02** | **10.94 (1.91-100.46)** | | **0.03** |  |
| ***Demographic data*** |  |  |  |  |  | |  |  |
| Female gender | 6 (54.6) | 176 (60.9) | 0.78 (0.23-2.58) | 0.6 |  | |  |  |
| Age, median (quartiles) | **84 (82-91)** | **75 (66.9-83.9)** | **…** | **0.001** |  | |  |  |
| Months since admission, median (quartiles) | 36 (18-48) | 48 (12-188) | … | 0.3 |  | |  |  |
| ***Medical data / comorbidities*** |  |  |  |  |  | |  |  |
| Karnofsky index, median (quartiles) | 40 (45-90) | 60 (55-70) | … | 0.1 |  | |  |  |
| Chalrson index, median (quartiles) | 2 (0-3) | 2 (1.5-2.5) | … | 0.8 |  | |  |  |
| Heart disease | 0 (0.0) | 27 (9.3) | 0.00 … | 0.3 |  | |  |  |
| Pulmonary disease | 0 (0.0) | 12 (4.2) | 0.00 … | 0.5 |  | |  |  |
| Renal disease | 0 (0.0) | 3 (1.0) | 0.00 … | 0.7 |  | |  |  |
| Diabetes mellitus | 0 (0.0) | 53 (18.3) | 0.00 … | 0.1 |  | |  |  |
| CNS disease (other than dementia) | 5 (45.5) | 115 (39.8) | 1.26 (0.38-4.23) | 0.7 |  | |  |  |
| Dementia | 7 (63.6) | 178 (61.6) | 1.09 (0.31-3.81) | 0.9 |  | |  |  |
| Solid malignancy | 2 (18.2) | 16 (5.5) | 3.79 (0.76-19.03) | 0.08 |  | |  |  |
| ***Exposure to healthcare**** |  |  |  |  |  | |  |  |
| Admission to acute care hospitals | **5 (45.5)** | **23 (8.0)** | **9.64 (2.73-34.61)** | **< 0.001** | **10.05 (2.52-40.06)** | | **0.001** |  |
| Surgery | 0 (0.0) | 15 (5.2) | 0.0 … | 0.4 |  | |  |  |
| Use of any antimicrobial | 3 (27.3) | 64 (22.15) | 1.31 (0.34-5.11) | 0.7 |  | |  |  |
| Use of β-lactams | 1 (9.1) | 32 (11.1) | 0.80 (0.10-6.48) | 0.8 |  | |  |  |
| Use of Quinolones | 2 (18.2) | 26 (9.0) | 2.25 (0.46-10.96) | 0.3 |  | |  |  |
| Use of Macrolides | 0 (0.0) | 11 (3.8) | 0.0 … | 0.5 |  | |  |  |
| Use of other antimicrobials | 1 (9.1) | 12 (4.5) | 2.30 (0.27-19.53) | 0.4 |  | |  |  |
| Naso-enteral tube feeding | 1 (9.1) | 7 (2.4) | 4.03 (0.45-35.93) | 0.2 |  | |  |  |
| Urinary catheter | 1 (9.1) | 13 (4.5) | 1.89 (0.23-15.81) | 0.4 |  | |  |  |
| ***Recent infections**** |  |  |  |  |  | |  |  |
| Skin/Soft tissue | 1 (9.1) | 37 (12.8) | 0.68 (0.09-5.48) | 0.7 |  | |  |  |
| Pneumonia | 1 (9.1) | 18 (6.2) | 1.51 (0.18-12.42) | 0.7 |  | |  |  |

**Note.** Data in number(%), except when specified. Significant results are presented in boldface. MRSA, methicillin-resistant *Staphylococcus aureus.*

OR, Odds Ratio. CI, Confidence Interval, CNS, Central Nervous System.

*In the past year.
